# Current Smoking Dose-Dependently Associated with Decreased *β*-Cell Function in Chinese Men without Diabetes

**DOI:** 10.1155/2015/841768

**Published:** 2015-07-05

**Authors:** Chun Wang, Yijun Wang, Junxia Wu, Suyi Liu, Ying Zhu, Shurong Lv, Ping Lin, Xiaoke Wang, Yan Xu, Shali Yu, Gang Chen, Quanyong Xiang

**Affiliations:** ^1^Department of Environmental Health, School of Public Health, Nantong University, Nantong, Jiangsu 226000, China; ^2^Department of Comprehensive Statistics, The Third People's Hospital of Nantong, Nantong, Jiangsu 226000, China; ^3^Department of Computer Science, School of Computer Science and Technology, Nantong University, Nantong, Jiangsu 226000, China; ^4^Department of Chronic Non-Communicable Disease Control, Jiangsu Provincial Center for Disease Control and Prevention, Nanjing, Jiangsu 210009, China

## Abstract

The aim of this study was to evaluate the associations between chronic smoking and insulin resistance and *β*-cell function in Chinese men without diabetes. A total of 1,568 participants were recruited by multistage sampling. Using homeostatic model assessment (HOMA), geometric means of insulin resistance (HOMA-IR) and *β*-cell function (HOMA-*β*) with 95% confidence interval (CI) were calculated by general linear model. Odds ratios (ORs) with 95% CI were estimated to evaluate the associations between smoking status and insulin resistance and *β*-cell deficiency under a logistic regression model. Current smokers had higher levels of 2 h glucose (6.66 versus 6.48 mmol/L) for oral glucose tolerance test and lower levels of fasting insulin (5.68 versus 6.03 mU/L) than never smokers. The adjusted means for HOMA-*β* (%) were 54.86 in current smokers and 58.81 in never smokers (*P* = 0.0257). Current smoking was associated with *β*-cell deficiency (OR 1.29, 95% CI 1.01–1.64) compared to never smoking. The *β*-cell function gradually decreased with increasing smoking intensity (*P*_trend_ = 0.0026), and the differences were statistically significant when the pack-year of smoking was 20 or above. No association was observed between smoking status and HOMA-IR. Our study suggested that chronic smoking may dose-dependently suppress insulin secretion in Chinese men.

## 1. Introduction

Tobacco use has fallen in many high-income countries, at least in men, but is now rising rapidly in many low- and middle-income countries such as China [1]. Thirty-eight percent of the world's smokers are Chinese, especially Chinese men who smoke one-third of the world's cigarettes, with 52.9% smoking prevalence and 50.4% current cigarette use [2, 3]. Tobacco use has become the number one killer in China and is responsible for 1.2 million deaths annually, and this number is expected to rise to 3.5 million deaths annually by the year 2030 [2]. Diabetes is another major public health problem in China. According to 2010 China Noncommunicable Disease Surveillance, the overall prevalence of diabetes and prediabetes was estimated to be 11.6% (12.1% among men) and 50.1% (52.1% among men) in Chinese adults, respectively, representing up to 113.9 million adults with diabetes and 493.4 million with prediabetes [4]. Furthermore, the current prevalence of diabetes in the Chinese population is very similar to the US population even though overweight and obesity are much more common in the latter [4]. It is of urgent need to illuminate associated risk factors so as to develop effective strategies to prevent the development of diabetes in China.

The association between smoking and increased risk for Type 2 diabetes mellitus (Type 2 DM) has been well documented [5, 6]. Both active and passive smoking have been demonstrated to be associated with an increased risk of Type 2 DM [7, 8]. Recent study also reported that current smokers were dose-dependently associated with increased risk for incident metabolic syndrome in Chinese men [9]. Insulin resistance (IR) and declining *β*-cell function have proven to be potential mechanisms in the development of Type 2 DM and can be demonstrated long before overt diabetes is diagnosed [10, 11]. Chronic smoking was reported to markedly and in a dose-dependent manner aggravate IR observed in Type 2 DM patients [12]. However, when the metabolic effects of smoking, especially chronic smoking, were studied in persons without diabetes, the findings were controversial. Smoking was reported to acutely impair insulin action and led to IR in normal subjects [13]. Chronic smoking was also shown to be associated with IR and its degree [5, 14–17]. Nevertheless, some studies found no difference in IR between smokers and nonsmokers in population without diabetes [18–23]. On the other hand, the effect of smoking on *β*-cell function was little explored. Few available studies failed to provide conclusive evidence [18, 19, 23]. Even opposite effects on *β*-cell function were reported in current and former smokers, when compared with nonsmokers [17].

Homeostatic model assessment (HOMA) using steady-state glucose and insulin is a method for assessing IR and *β*-cell function and is more appropriate for use in large epidemiological studies [24]. But few studies applying HOMA method are currently available for assessing the associations of chronic smoking in Asians who exhibit lower levels of the HOMA of insulin resistance (HOMA-IR) than western populations [17–19]. Moreover, a dynamic evaluation of *β*-cell function, such as oral glucose tolerance test (OGTT), according to smoking status was rare. This population-based study aims to elucidate the effects of chronic smoking on IR and *β*-cell function in a large sample of Chinese men without diabetes using both HOMA analysis and OGTT.

## 2. Subjects and Methods

### 2.1. Subjects

On the basis of China Noncommunicable Disease Surveillance in 2010, the study population in this cross-sectional study was a subset population recruited in Jiangsu Province, using a complex, multistage, probability sampling design [4]. In brief, we first selected 6 counties from 106 counties using stratified random sampling according to the population, gross domestic product (GDP), degree of urbanization, and geographic setting. Secondly, 4 towns from each county and 3 villages from each town were selected with the probability proportional to the population size, using cluster random sampling. Thirdly, 1 residential group including at least 50 households was selected from each village using simple random cluster sampling. Random digit function in Excel was applied in selection of these households from each residential group. Finally, one family member aged 18 years or over was randomly selected from each household using the KISH Grid method [25, 26]. The subjects who were diagnosed as diabetes according to the American Diabetes Association criteria were excluded from this study [27]. A total of 1,568 men without diabetes were included in the present study. This study was approved by the Institutional Review Board. A written informed consent was obtained from each participant.

### 2.2. Collection of Epidemiological Data

Epidemiological information was collected through a standardized questionnaire by the trained interviewers. The data collected during the face-to-face interview included demographic characteristics, lifestyle factors, and medical history. The investigated smoking history included age at smoking initiation, years of smoking, number of cigarettes smoked per day, and smoking cessation. The smoking status was defined as previously described [28]. Briefly, an individual who never smoked or smoked less than 100 cigarettes in his lifetime was defined as a never smoker. An individual who smoked at least 100 cigarettes in his lifetime but quit smoking more than 12 months before the interview was considered as a former smoker. Current smokers included those currently smoking and those who quit smoking less than 12 months before interview. The pack-year of smoking was calculated according to the number of packs of cigarettes smoked per day and smoking duration (years). Information about the amount and type of consumed alcohol was collected as well. An individual who never consumed alcohol or consumed less than or equal to one drink per month was defined as a never drinker, otherwise as an ever drinker. The Global Physical Activity Questionnaire was used to evaluate physical activities by calculating the total weekly volume (metabolic equivalents (MET) min/wk) across three separate domains (work/home, during commuting, and during leisure time), by applying MET values to the time variables according to the intensity of the activity [4, 29, 30].

### 2.3. Measurement of Anthropometric Index and Blood Pressure

Anthropometric index included height, body weight, and waist circumference. Height and weight were measured in light underclothes without shoes. In the standing position of participants in light clothing, waist circumference was measured at the midway between the lower edge of the costal arch and the upper edge of the iliac crest. Body mass index (BMI) was calculated as weight (kg)/height (m^2^). BMI ≥ 25 kg/m^2^ was considered as overweight, and waist circumference ≥ 90 cm as central obesity for Chinese men [4, 31].

Blood pressure was measured by trained physicians using calibrated electronic sphygmomanometer (HEM-7071, Omron Corporation, Japan), on the nondominant arm in precordial level after at least 5 min of rest and with the subjects in sitting position. Three readings were taken 2 min apart and the averaged values of systolic blood pressure (SBP) and diastolic blood pressure (DBP) were calculated. The participants were identified as hypertensive subjects if they had high blood pressure values of SBP ≥ 140 mmHg and/or DBP ≥ 90 mmHg or if they had a history of hypertension or if they were under antihypertensive medications therapy.

### 2.4. Measurement of Glucose, Insulin, and Lipids in Blood

A venous blood sample (4-5 mL) was drawn from each participant after overnight fasting of at least 10 hours. Plasma samples for glucose test were collected with the tubes containing sodium fluoride at 0 h and 2 h after administration of a standard 75 g glucose solution during OGTT test. Plasma glucose was measured using glucose oxidase or hexokinase methods within 24 h. Capillary blood samples for glycated hemoglobin (HbA1c) detection were collected by The Hemoglobin Capillary Collection System. HbA1c was measured within 4 w by high-performance liquid chromatography using the VARIANT II Hemoglobin Testing System (Bio-Rad Laboratories). Capillary HbA1c was converted to venous values using a validated formula [4]. Serum specimens were collected for detection of insulin and lipids. Serum insulin was measured by the electrochemiluminescence immunoassay on an automatic electrochemiluminescence analyzer (COBAS-E601, Roche Company). Serum total cholesterol (TC), triglyceride (TG), low density lipoprotein cholesterol (LDL), and high density lipoprotein cholesterol (HDL) were detected with the enzymatic methods using an automatic biochemistry analyzer (Abbott Laboratories) [4].

On the basis of fasting glucose and insulin levels, insulin resistance and secretion were evaluated using HOMA method with the formulas as HOMA-IR = fasting insulin (mU/L) × fasting glucose (mmol/L)/22.5 and *β*-cell function (HOMA-*β*) % = [20 × fasting insulin (mU/L)]/[fasting glucose (mmol/L) − 3.5] [24]. Individuals with fasting glucose of 3.5 mmol/L or less were excluded from analysis. The cutoff points for IR and *β*-cell deficiency were HOMA-IR ≥ 2.6 and HOMA-*β* (%) < 50, respectively [24, 32]. Dyslipidemia was defined by the presence of at least one abnormal serum lipid: TC ≥ 5.18 mmol/L, TG ≥ 1.7 mmol/L, LDL ≥ 3.37 mmol/L, and HDL in men < 1.04 mmol/L [31].

### 2.5. Statistical Analyses

All the data were double entered in the database and corrected for errors using Epi-info 6.0 software. The statistical analyses were performed using the SAS software version 9.2 (SAS institute, Cary, NC). The differences in the distributions of demographic characteristics among never, former, and current smokers were compared by an unpaired Student's* t*-test for continuous variables and a *χ* ^2^ test for categorical variables, using never smokers as references. To improve skewness, fasting insulin, glucose, HbA1c, HOMA-IR, and HOMA-*β* were logarithmically transformed for statistical analyses. Unadjusted and adjusted geometric means (adjustment for age, education level, drinking status, BMI, waist circumference, level of physical activity, hypertension, and dyslipidemia) with 95% confidence interval (CI) were calculated by general linear model and back transformed to natural units for presentation. The associations between smoking status and IR and *β*-cell deficiency were evaluated under univariate and multivariate unconditional logistic regression model after adjustment for covariates. Odds ratio (OR) with 95% CI was used to estimate the strength of association. Dose-dependent effect of smoking intensity on *β*-cell function was also analyzed as a continuous variable using fractional polynomial regression model. All the statistical tests were two-sided, and *P* < 0.05 was considered as statistically significant.

## 3. Results

### 3.1. General Characteristics of the Study Population

The 1,568 Chinese men without diabetes in this study included 598 never smokers, 120 former smokers, and 850 current smokers (Table 1). Most of the smokers (93.71%) smoked manufactured cigarettes. There was no significant difference in age between current and never smokers; however, the former smokers were younger than never smokers (*P* = 0.0430). Higher percentages of ever drinkers were observed in both current (72.35%) and former (75.83%) smokers, compared with the percentage of 55.52% in never smokers (*P* < 0.0001). Moreover, current smokers with a median equivalent combination of physical activity of 3360 MET min/wk were more physically active, compared to never smokers (2520 MET min/wk, *P* = 0.0063). Although there were more subjects with hypertension and dyslipidemia in current smokers, and more participants who had a higher level of education, BMI, and waist circumference in former smokers, the differences were not statistically significant.

### 3.2. *β*-Cell Function and Insulin Resistance by Smoking Status

There was no statistical difference in fasting glucose; however, the levels of 2 h glucose for OGTT were significantly higher in current smokers than in never smokers (*P* = 0.0493) (Table 2). Compared to never smokers, current smokers had significantly decreased fasting insulin, after adjustment for covariates (*P* = 0.0335). The adjusted means with 95% CI for HOMA-*β* (%) were 54.86 (52.10–57.78) in current smokers and 58.81 (55.57–62.24) in never smokers (*P* = 0.0257). No significant difference in HbA1c and HOMA-IR was observed when comparing current or former smoker with never smokers.

### 3.3. Associations between Smoking Status and *β*-Cell Deficiency

As shown in Table 3, there were 57.42% current smokers with a HOMA-*β* value less than 50. Current smoking was associated with *β*-cell deficiency (OR 1.23, 95% CI 1.00–1.52), compared to never smoking. The association was still statistically significant (OR 1.29, 95% CI 1.01–1.64) after adjustment for age, education level, drinking status, BMI, waist circumference, level of physical activity, hypertension, and dyslipidemia. No association was observed between former smoking and *β*-cell deficiency, as well as between smoking status and HOMA-IR.

**Figure 1:**
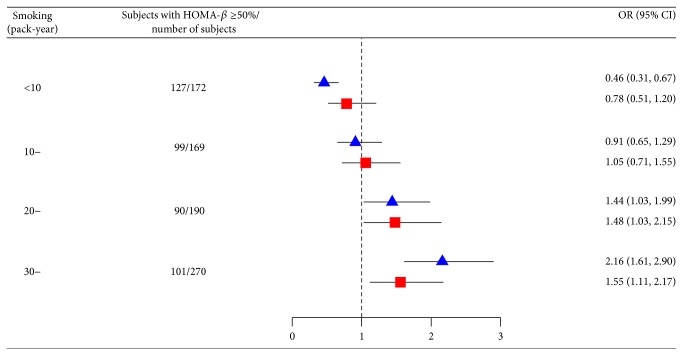
The associations between smoking intensity and *β*-cell function. The triangles and squares represent crude odds ratios (ORs) and ORs after adjustment for age, education level, drinking status, body mass index, waist circumference, level of physical activity, hypertension, and dyslipidemia. The horizontal lines represent 95% confidence interval.

**Figure 2:**
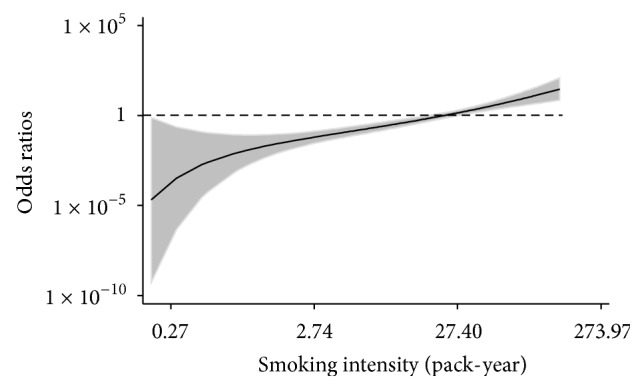
Dose-dependent effect of smoking intensity on *β*-cell deficiency. Fractional polynomial regression model is used to analyze the association between smoking intensity and *β*-cell deficiency. Solid line represents odds ratios; shaded area shows 95% confidence interval; dash line indicates the reference.

The strength of association with *β*-cell deficiency was further analyzed after categorizing current smokers according to their pack-year of smoking and using never smokers as references. A dose-dependent effect of smoking intensity was observed to be associated with impaired *β*-cell function, however, not with HOMA-IR. The *β*-cell function gradually decreased with increasing smoking intensity (*P*_trend_ < 0.0001 and 0.0026 after adjustment for covariates), and the differences were statistically significant when the pack-year of smoking was 20 or above (Figure 1). To evaluate the observed association between smoking intensity and *β*-cell function in a more dynamic manner, we further analyzed the dose-dependent effect of smoking intensity as a continuous variable on *β*-cell deficiency using fractional polynomial regression model. A significant linear trend of increasing ORs along with increasing smoking intensity was observed (Figure 2, *P*_trend_ < 0.0001), which was consistent with the results under logistic regression analysis of smoking intensity as a categorical variable. In addition, fasting glucose increased with cumulative intensity of smoking; the adjusted means (95% CI) were 5.65 (5.56–5.74), 5.58 (5.44–5.73) (*P* = 0.3857), 5.55 (5.42–5.69) (*P* = 0.1806), 5.72 (5.59–5.86) (*P* = 0.3271), and 5.78 (5.66–5.91) (*P* = 0.0384), respectively, for never smokers and current smokers with the pack-year of smoking <10, 10~, 20~, and ≥30 (*P*_trend_ = 0.2177).

## 4. Discussion

In this population-based study, we evaluated the associations between chronic smoking and IR and *β*-cell function in Chinese men without diabetes. Our results showed that current smoking was associated with decreased *β*-cell function in a dose-dependent manner, and the levels of 2 h glucose were significantly higher in current smokers than in never smokers.

The metabolic effects of smoking have generally been studied in persons without diabetes, in order to avoid the impacts from the medications or changed behaviors in patients with diabetes [5]. IR and *β*-cell function are usually determined by the hyperinsulinemic-euglycemic clamp and the hyperglycemic clamp [24]. Using the euglycemic clamp technique, Eliasson et al. [14, 33] observed IR in middle-aged men who chronically smoke and its normalization eight weeks after smoking cessation. However, in the Insulin Resistance Atherosclerosis Study, smoking was not associated with IR as assessed by a modified glucose tolerance test with minimal model analysis [22]. Although referred to as the gold standard tests, clamps are complex stress tests with insulin and glucose concentrations and flux well outside the normal range [24]. Moreover, clamp techniques are only suitable to be applied in studies with relatively small numbers of subjects. Alternately, HOMA, a method for assessing IR and *β*-cell function from basal glucose and insulin concentration, correlates well with estimates using other methods including clamps and is more appropriate for use in large epidemiological studies [24]. The relationship between glucose and insulin in the basal state reflects the balance between hepatic glucose output and insulin secretion, which is maintained by a feedback loop between the liver and *β*-cells [34]. But recent studies using the HOMA method reported inconsistent effects of smoking in Caucasians, such as high *β*-cell values and IR in normoglycaemic persons [17], lower *β*-cell function in men without hypertension and diabetes [18], or no association in men without diabetes [19]. The study of Ko et al. [23] in Chinese men without diabetes showed that no statistical difference in HOMA-IR or HOMA-*β* was observed among current, former, and never smokers. But in this study smokers merely accounted for 22.53% (178 out of 790) of subjects without diabetes, and men with newly diagnosed impaired glucose tolerance (IGT) were excluded. Our study including 1,568 men without diabetes found impaired *β*-cell secretion in current smokers, which was signified by both low values of HOMA-*β* and fasting insulin. The inconsistency among these studies using HOMA method may be attributable to the differences in study design, subject recruit, ethnic origin, gender stratification, involved covariates, and sample size.

Nicotine is the critical substance which exerts most effects of smoking. Animal experiments showed that both prenatal and postnatal exposure to nicotine could directly induce imbalance of metabolic control [35]. The studies using rodent models demonstrated that nicotine exposure could cause *β*-cell dysfunction, elevated pancreatic *β*-cell apoptosis, and loss of *β*-cell mass, which was mediated via the mitochondrial and/or death receptor pathway [36]. Smoking cessation could possibly reverse the unfavorable effects from nicotine. A recent study by Stadler et al. [37] reported that smoking cessation was associated with metabolic changes including increased *β*-cell secretion in response to glucose. All these findings provided consistent evidence and biological plausibility for the decreased insulin secretion in smokers, especially heavy smokers in our study. In the dynamic evaluation of *β*-cell function using OGTT test, we also found higher levels of 2 h glucose in current smokers, which may be attributed to long-term effects of nicotine exposure or may be from acute stress due to nicotine withdrawal [38].

Either acute or chronic nicotine exposure was reported to negatively affect insulin action in smokers preceded before Type 2 DM [35]. Long-term use of nicotine-containing chewing gum in nonsmoking men was associated with IR and hyperinsulinemia [39]. However, no association was observed between smoking and HOMA-IR in the current study. It is noteworthy that HOMA-IR, based on fasting insulin and glucose measurements to determine whole-body IR, primarily reflects hepatic (central) insulin sensitivity rather than impaired glucose uptake and consumption in skeletal muscle and adipose tissue (peripheral), thus limiting its ability to assess IR, especially in individuals with IGT [40–43]. It was suggested that insulin responses during the OGTT may be a better surrogate measure for IR, particularly those that occurred in peripheral insulin-sensitive tissues such as muscle [42, 44, 45]. Pisprasert et al. [41] also pointed out that none of the surrogate estimates of insulin sensitivity in their study was superior to simple measurement of fasting plasma insulin concentrations in predicting insulin sensitivity. In our study, increased 2 h plasma glucose during OGTT test indicated probably insulin resistance in current smokers.

In the present study based on the national survey, the subjects recruited using random sampling methods were representative. OGTT was used to dynamically evaluate *β*-cell function according to smoking status. Physical activities were investigated using standard questionnaires and evaluated by metabolic equivalent-minutes per week. However, there were some limitations in our study. First, we only detected 2 h plasma glucose after administration of a standard glucose solution. Area under the curve which is more important in dynamic measurement could not be calculated and compared in this study. Second, due to few former smokers available, we did not further analyze when *β*-cell dysfunction in smokers would return to normal after smoking cessation. Moreover, passive smoking was not investigated. Third, our study lacked information on other major confounders such as socioeconomic factors, psychosocial stress, and dietary patterns. Fourth, the participants in this study were Chinese men, which restricted the generalization of our results to other ethnicities and gender. Last, cross-sectional design could not provide a causal relationship between smoking and *β*-cell deficiency. Therefore, large-scale prospective studies are warranted to confirm our findings.

In conclusion, our study suggested that chronic smoking impaired insulin secretion and probably brought about insulin resistance in Chinese men without diabetes. Taking into consideration the increased risk of development of Type 2 DM related to *β*-cell dysfunction in current smokers, tobacco control should be considered the most urgent and immediate priority in China, especially in Chinese men [1].

## Figures and Tables

**Table 1:** General characteristics of study population.

Variables	Never smokers	Former smokers		Current smokers	
	(*N* = 598, %)	(*N* = 120, %)	*P* ^*∗*^	(*N* = 850, %)	*P* ^*∗*^
Age (y), mean (SD)	52.03 (15.68)	48.89 (14.31)	0.0430	50.54 (13.69)	0.0615
Education level					
Primary school or lower	213 (35.62)	33 (27.50)	0.0872	310 (36.47)	0.7397
Middle school or higher	385 (64.38)	87 (72.50)		540 (63.53)	
Drinking status					
Never	266 (44.48)	29 (24.17)	<0.0001	235 (27.65)	<0.0001
Ever	332 (55.52)	91 (75.83)		615 (72.35)	
Body mass index (kg/m^2^)					
<25	343 (57.36)	59 (49.17)	0.0990	508 (59.76)	0.3596
≥25	255 (42.64)	61 (50.83)		342 (40.24)	
Waist circumference (cm)					
<90	401 (67.06)	72 (60.00)	0.1368	581 (68.35)	0.6032
≥90	197 (32.94)	48 (40.00)		269 (31.65)	
Physical activity (MET min/wk)					
≥2520	273 (45.65)	52 (43.33)	0.8858	430 (50.59)	0.0154
<2520	265 (44.31)	56 (46.67)		314 (36.94)	
Unknown	60 (10.03)	12 (10.00)		106 (12.47)	
Hypertension					
No	279 (46.66)	57 (47.50)	0.8656	382 (44.94)	0.5190
Yes	319 (53.34)	63 (52.50)		468 (55.06)	
Dyslipidemia					
No	426 (71.24)	78 (65.00)	0.1728	585 (68.82)	0.3245
Yes	172 (28.76)	42 (35.00)		265 (31.18)	

SD: standard deviation; MET: metabolic equivalent.

^*∗*^ *P* values were calculated using never smokers as references.

**Table 2:** *β*-cell function and insulin resistance by smoking status.

Variables	Never smokers			Former smokers			Current smokers		
	*N*	Mean (95% CI)	Mean (95% CI)^*∗*^	*N*	Mean (95% CI)	Mean (95% CI)^*∗*^	*N*	Mean (95% CI)	Mean (95% CI)^*∗*^
Fasting glucose (mmol/L)	598	5.60 (5.53–5.67)	5.66 (5.58–5.75)	119	5.56 (5.41–5.72)	5.60 (5.44–5.76)	849	5.63 (5.57–5.69)	5.69 (5.61–5.77)
					*P* = 0.6660	*P* ^*∗*^ = 0.4562		*P* = 0.5119	*P* ^*∗*^ = 0.5712
2 h glucose (mmol/L)	569	6.32 (6.18–6.47)	6.48 (6.29–6.67)	112	6.19 (5.82–6.59)	6.36 (5.98–6.76)	794	6.56 (6.38–6.74)	6.66 (6.45–6.87)
					*P* = 0.5417	*P* ^*∗*^ = 0.5576		*P* = 0.0493	*P* ^*∗*^ = 0.1244
Fasting insulin (mU/L)	598	5.43 (5.15–5.72)	6.03 (5.73–6.34)	120	5.40 (4.80–6.08)	5.59 (5.08–6.16)	849	5.12 (4.89–5.35)	5.68 (5.42–5.95)
					*P* = 0.9430	*P* ^*∗*^ = 0.1502		*P* = 0.0953	*P* ^*∗*^ = 0.0335
HbA1c (%)	593	5.79 (5.74–5.83)	5.80 (5.75–5.86)	120	5.75 (5.65–5.85)	5.79 (5.69–5.89)	848	5.80 (5.76–5.83)	5.83 (5.78–5.88)
					*P* = 0.4706	*P* ^*∗*^ = 0.7518		*P* = 0.7671	*P* ^*∗*^ = 0.3662
HOMA-IR	598	1.35 (1.27–1.43)	1.52 (1.43–1.60)	119	1.34 (1.18–1.52)	1.39 (1.25–1.55)	848	1.28 (1.22–1.34)	1.44 (1.36–1.51)
					*P* = 0.8959	*P* ^*∗*^ = 0.1388		*P* = 0.1675	*P* ^*∗*^ = 0.0723
HOMA-*β* (%)	598	54.39 (51.57–57.37)	58.81 (55.57–62.24)	119	54.70 (48.54–61.64)	55.90 (50.16–62.29)	848	50.60 (48.39–52.92)	54.86 (52.10–57.78)
					*P* = 0.9334	*P* ^*∗*^ = 0.3813		*P* = 0.0420	*P* ^*∗*^ = 0.0257

HbA1c, glycated hemoglobin; HOMA-IR, homeostasis model assessment of insulin resistance; HOMA-*β*, homeostasis model assessment of *β*-cell function; CI, confidence interval.

^*∗*^Adjusted for age, education level, drinking status, body mass index, waist circumference, level of physical activity, hypertension, and dyslipidemia, using never smokers as references.

**Table 3:** The associations between smoking status and *β*-cell deficiency and insulin resistance.

Smoking status	*β*-cell deficiency				Insulin resistance			
	No	Yes	OR (95% CI)	OR (95% CI)^*∗*^	No	Yes	OR (95% CI)	OR (95% CI)^*∗*^
Never	337 (39.93)	261 (36.20)	1.00	1.00	505 (38.37)	93 (37.35)	1.00	1.00
Former	73 (8.65)	46 (6.38)	0.81 (0.54–1.22)	0.94 (0.59–1.49)	99 (7.52)	20 (8.03)	1.10 (0.65–1.86)	0.77 (0.42–1.41)
Current	434 (51.42)	414 (57.42)	1.23 (1.00–1.52)	1.29 (1.01–1.64)	712 (54.10)	136 (54.62)	1.04 (0.78–1.38)	0.95 (0.68–1.32)

OR, odds ratio; CI, confidence interval.

^*∗*^Adjusted for age, education level, drinking status, body mass index, waist circumference, level of physical activity, hypertension, and dyslipidemia.

## References

[1] Beaglehole R., Bonita R., Horton R. (2011). Priority actions for the non-communicable disease crisis. *The Lancet*.

[2] Eriksen M., Mackay J., Ross H. (2012). *The Tobacco Atlas*.

[3] Chinese Center for Disease Control and Prevention Global Adult Tobacco Survey (GATS) China 2010 Country Report. http://www.who.int/tobacco/surveillance/survey/gats/en_gats_china_report.pdf.

[4] Xu Y., Wang L., He J. (2013). Prevalence and control of diabetes in Chinese adults. *The Journal of the American Medical Association*.

[5] Centers for Disease Control and Prevention (US). National Center for Chronic Disease Prevention and Health Promotion (US). Office on Smoking and Health (US) (2010). *How Tobacco Smoke Causes Disease: The Biology and Behavioral Basis for Smoking-Attributable Disease: A Report of the Surgeon General*.

[6] Clair C., Cornuz J. (2010). Diabetes: risk of diabetes mellitus: should smokers quit smoking?. *Nature Reviews Endocrinology*.

[7] Willi C., Bodenmann P., Ghali W. A., Faris P. D., Cornuz J. (2007). Active smoking and the risk of type 2 diabetes: a systematic review and meta-analysis. *The Journal of the American Medical Association*.

[8] Wang Y., Ji J., Liu Y.-J., Deng X., He Q.-Q. (2013). Passive smoking and risk of type 2 diabetes: a meta-analysis of prospective cohort studies. *PLoS ONE*.

[9] Zhu Y., Zhang M., Hou X. (2011). Cigarette smoking increases risk for incident metabolic syndrome in Chinese men-Shanghai diabetes study. *Biomedical and Environmental Sciences*.

[10] Kahn S. E. (2003). The relative contributions of insulin resistance and beta-cell dysfunction to the pathophysiology of Type 2 diabetes. *Diabetologia*.

[11] Ndisang J. F., Rastogi S., Vannacci A. (2014). Insulin resistance, type 1 and type 2 diabetes, and related complications: current status and future perspective. *Journal of Diabetes Research*.

[12] Targher G., Alberiche M., Zenere M. B., Bonadonna R. C., Muggeo M., Bonora E. (1997). Cigarette smoking and insulin resistance in patients with noninsulin—dependent diabetes mellitus. *Journal of Clinical Endocrinology and Metabolism*.

[13] Attvall S., Fowelin J., Lager I., Von Schenck H., Smith U. (1993). Smoking induces insulin resistance—a potential link with the insulin resistance syndrome. *Journal of Internal Medicine*.

[14] Eliasson B., Attvall S., Taskinen M.-R., Smith U. (1994). The insulin resistance syndrome in smokers is related to smoking habits. *Arteriosclerosis and Thrombosis*.

[15] Facchini F. S., Hollenbeck C. B., Jeppesen J., Chen Y.-D. I., Reaven G. M. (1992). Insulin resistance and cigarette smoking. *The Lancet*.

[16] Reaven G. M., Ida Chen Y.-D. (1992). Insulin resistance and cigarette smoking. *The Lancet*.

[17] Daniel M., Cargo M. D. (2004). Association between smoking, insulin resistance and beta-cell function in a North-western First Nation. *Diabetic Medicine*.

[18] Östgren C. J., Lindblad U., Ranstam J., Melander A., Råstam L. (2000). Associations between smoking and *β*-cell function in a non-hypertensive and non-diabetic population. *Diabetic Medicine*.

[19] Masulli M., Riccardi G., Galasso R., Vaccaro O. (2006). Relationship between smoking habits and the features of the metabolic syndrome in a non-diabetic population. *Nutrition, Metabolism and Cardiovascular Diseases*.

[20] Wareham N. J., Ness E. M., Byrne C. D., Cox B. D., Day N. E., Hales C. N. (1996). Cigarette smoking is not associated with hyperinsulinemia: evidence against a causal relationship between smoking and insulin resistance. *Metabolism: Clinical and Experimental*.

[21] Godsland I. F., Walton C. (1992). Insulin resistance and cigarette smoking. *The Lancet*.

[22] Henkin L., Zaccaro D., Haffner S. (1999). Cigarette smoking, environmental tobacco smoke exposure and insulin sensitivity: the Insulin Resistance Atherosclerosis Study. *Annals of Epidemiology*.

[23] Ko G. T.-C., Tong P. C.-Y., So W.-Y., Cockram C. S., Chan J. C.-N. (2007). Association between smoking, pancreatic insulin secretion and insulin resistance in Chinese subjects with or without glucose intolerance. *Chinese Medical Journal*.

[24] Wallace T. M., Levy J. C., Matthews D. R. (2004). Use and abuse of HOMA modeling. *Diabetes Care*.

[25] Smith W., Chey T., Jalaludin B., Salkeld G., Capon T. (1995). Increasing response rates in telephone surveys: a randomized trial. *Journal of Public Health Medicine*.

[26] Li Y., Dong X., Wang S. (2013). Consciousness and abilities on health emergency and the roles of emergency response among public at the communities. *Zhonghua Liu Xing Bing Xue Za Zhi*.

[27] American Diabetes Association (2010). Diagnosis and classification of diabetes mellitus. *Diabetes Care*.

[28] Spitz M. R., Hong W. K., Amos C. I. (2007). A risk model for prediction of lung cancer. *Journal of the National Cancer Institute*.

[29] Armstrong T., Bull F. (2006). Development of the World Health Organization Global Physical Activity Questionnaire (GPAQ). *Journal of Public Health*.

[30] Lee J. M., Woolford S., Herman W. H., Clark S. J. (2010). Does childhood overweight, parental perception of overweight, or family history of diabetes mellitus increase parental perception of type 2 diabetes risk for their child?. *Journal of Pediatric Endocrinology and Metabolism*.

[31] Du S.-M., Ma G.-S., Li Y.-P. (2010). Relationship of body mass index, waist circumference and cardiovascular risk factors in Chinese adult. *Biomedical and Environmental Sciences*.

[32] Ascaso J. F., Pardo S., Real J. T., Lorente R. I., Priego A., Carmena R. (2003). Diagnosing insulin resistance by simple quantitative methods in subjects with normal glucose metabolism. *Diabetes Care*.

[33] Eliasson B., Attvall S., Taskinen M.-R., Smith U. (1997). Smoking cessation improves insulin sensitivity in healthy middle-aged men. *European Journal of Clinical Investigation*.

[34] Turner R. C., Holman R. R., Matthews D., Hockaday T. D. R., Peto J. (1979). Insulin deficiency and insulin resistance interaction in diabetes: estimation of their relative contribution by feedback analysis from basal plasma insulin and glucose concentrations. *Metabolism*.

[35] Xie X.-T., Liu Q., Wu J., Wakui M. (2009). Impact of cigarette smoking in type 2 diabetes development. *Acta Pharmacologica Sinica*.

[36] Bruin J. E., Gerstein H. C., Morrison K. M., Holloway A. C. (2008). Increased pancreatic beta-cell apoptosis following fetal and neonatal exposure to nicotine is mediated via the mitochondria. *Toxicological Sciences*.

[37] Stadler M., Tomann L., Storka A. (2014). Effects of smoking cessation on *β*-cell function, insulin sensitivity, body weight, and appetite. *European Journal of Endocrinology*.

[38] Wardle M. C., Munafò M. R., de Wit H. (2011). Effect of social stress during acute nicotine abstinence. *Psychopharmacology*.

[39] Eliasson B., Taskinen M.-R., Smith U. (1996). Long-term use of nicotine gum is associated with hyperinsulinemia and insulin resistance. *Circulation*.

[40] Reaven G. M. (2013). What do we learn from measurements of HOMA-IR?. *Diabetologia*.

[41] Pisprasert V., Ingram K. H., Lopez-Davila M. F., Munoz A. J., Garvey W. T. (2013). Limitations in the use of indices using glucose and insulin levels to predict insulin sensitivity: impact of race and gender and superiority of the indices derived from oral glucose tolerance test in African Americans. *Diabetes Care*.

[42] Ferrara C. M., Goldberg A. P. (2001). Limited value of the homeostasis model assessment to predict insulin resistance in older men with impaired glucose tolerance. *Diabetes Care*.

[43] Yeni-Komshian H., Carantoni M., Abbasi F., Reaven G. M. (2000). Relationship between several surrogate estimates of insulin resistance and quantification of insulin-mediated glucose disposal in 490 healthy nondiabetic volunteers. *Diabetes Care*.

[44] Abdul-Ghani M. A., Matsuda M., Balas B., DeFronzo R. A. (2007). Muscle and liver insulin resistance indexes derived from the oral glucose tolerance test. *Diabetes Care*.

[45] Nathan D. M., Davidson M. B., DeFronzo R. A. (2007). Impaired fasting glucose and impaired glucose tolerance: implications for care. *Diabetes Care*.

